# The Role of Mitochondria Dysfunction in Inflammatory Bowel Diseases and Colorectal Cancer

**DOI:** 10.3390/ijms222111673

**Published:** 2021-10-28

**Authors:** Patrycja Kłos, Siarhei A. Dabravolski

**Affiliations:** 1 Department of Biochemistry and Medical Chemistry, Pomeranian Medical University, 72 Al. Powstańców Wlkp., 70-111 Szczecin, Poland; patison@pum.edu.pl; 2 Department of Clinical Diagnostics, Vitebsk State Academy of Veterinary Medicine [UO VGAVM], 7/11 Dovatora Str., 210026 Vitebsk, Belarus

**Keywords:** inflammatory bowel diseases, colorectal cancer, chronic inflammation, mitochondrial dysfunction, mitochondrial mutations

## Abstract

Inflammatory bowel disease (IBD) is one of the leading gut chronic inflammation disorders, especially prevalent in Western countries. Recent research suggests that mitochondria play a crucial role in IBD development and progression to the more severe disease—colorectal cancer (CRC). In this review, we focus on the role of mitochondrial mutations and dysfunctions in IBD and CRC. In addition, main mitochondria-related molecular pathways involved in IBD to CRC transition are discussed. Additionally, recent publications dedicated to mitochondria-targeted therapeutic approaches to cure IBD and prevent CRC progression are discussed.

## 1. Introduction

### 1.1. IBD—Description, Prevalence, Pathophysiology

IBDs (inflammatory bowel diseases) are particularly troublesome medical conditions manifested by chronic, uncontrolled inflammation of the gut. These difficult-to-manage diseases, the most common of which are UC (ulcerative colitis) and CD (Crohn’s disease), have periods of spontaneous exacerbation and remission. Despite several common features, such as chronic inflammation or dysregulated inflammatory response of the immune system, UC and CD show differences in their clinical presentation [1].

Ulcerative colitis is a chronic inflammatory process in the lining of the large intestine, predominantly in the colon and rectum [2]. The lesions mainly affect the mucosa and submucosa and do not penetrate outside the intestine. Abdominal pain and mucous diarrhoea with an admixture of fresh blood and sometimes pus are the most common disease symptoms. Anaemia, chronic fatigue, weakness, and fever often appear in the course of the disease. Possible extraintestinal symptoms include skin problems (e.g., aphthous stomatitis, erythema nodosum) and arthritis [3,4].

Crohn’s disease can affect any part of the digestive tract, from the mouth to anus, but is most often located in the ileum or the initial part of the large intestine. Unlike in CD, the lesions are transmural and segmental [5]. The accompanying symptoms, such as postprandial pain, diarrhoea with mucous, fever and fatigue [6], vary in severity, and the chronic inflammatory process may finally lead to fistulas, abscesses, and even intestinal obstruction. Malabsorption resulting from the disease can contribute to significant weight loss, vitamin and mineral deficiencies, anaemia, and electrolyte disturbances. The disease may also be accompanied by conjunctivitis and skin and joint complications, similar to UC [4,7].

Inflammatory bowel diseases are most prevalent in Western developed countries. Approximately 2 million people in Europe suffer from these diseases [8], while in the US they affect about 1.6 million residents [9]. Over the last 20 years, however, there has been an increase in the number of IBD cases in developing countries in Eastern Europe, as well as in Africa, South America, and even Asia, suggesting that in addition to the genetic basis of IBD, the occurrence of the disease may also be determined by non-genetic factors [10]. Indeed, the pathogenesis of IBD has been proven to be multifactorial and resulting from a complex interplay of genetic background and environmental factors. The latter—by affecting the intestinal microbiome—weaken the tightness of the intestinal barrier, which in turn leads to improper activation of immune cells and, consequently, to symptoms typical of inflammatory bowel diseases [5].

The relationship between the incidence of inflammatory bowel disease and the genetic background has been well established. The risk of developing IBD is increased in children whose parents suffered from inflammatory bowel diseases, reaching up to 36% if both parents were diagnosed with IBD [2]. Of the two most commonly diagnosed inflammatory bowel diseases, the link between genetics and disease prevalence is stronger in Crohn’s disease compared to ulcerative colitis [11]. To date, GWAS (genome-wide association studies) have identified over 250 genetic IBD risk factors, the first of which were variants in NOD2 (nucleotide-binding oligomerization domain-containing protein 2). NOD2, a pattern recognition receptor, is found mainly in the cells of the innate immune system, as well as in intestinal epithelial cells, and is responsible for the binding of muramyl dipeptide, which is a component of the bacterial cell wall. Mutations in the gene of this protein, resulting in impaired recognition of bacterial cell wall components, are associated with increased susceptibility to developing CD [5]. Additionally to CD, NOD1 and NOD2 are involved in metabolism regulation and associated with several human metabolic diseases (such as diabetes, obesity, metabolic syndrome, and non-alcoholic fatty liver disease) [12]. In general, most of the genetic risk factors identified by GWAS are mutations in genes coding for proteins associated with autophagy and immune response, and 5% of them are functionally linked to the maintenance of mitochondrial homeostasis [13].

The environmental risk factors that may increase the incidence of IBD include, inter alia, consuming highly processed foods rich in saturated fatty acids and taking medications, especially antibiotics and non-steroidal anti-inflammatory drugs [5]. Most, if not all, of these factors may change the composition of the gut flora, shifting the balance towards bacteria with pro-inflammatory capacities. This, in turn, can result in damage to the intestinal barrier and the development of inflammation in the intestinal mucosa [11,14]. Gut microbiota composition could also directly affect mitochondria of intestinal cells and promote IBD or CRC development [15].

The intestinal barrier formed by intestinal epithelial cells (enterocytes, goblet cells, neuroendocrine cells, PC (Paneth cells), and M cells) and cells of the innate immune system maintains the balance between the intestinal mucosa and the content of the intestinal lumen. This phenomenon involves the formation of a physical barrier (tight junctions between the cells), mucus production (Goblet cells), maintaining the homeostasis of the intestinal crypts, and secretion of antibacterial substances (Paneth cells). A reduction in the tightness of the intestinal barrier has been observed in patients suffering from CD and in some of their first-degree relatives, and intestinal biopsies collected from these patients have revealed the down-regulation of epithelial cadherin, which is involved in the formation of tight junctions [11]. Since the multidimensional process of maintaining the intestinal barrier is energy-consuming, a hypothesis has emerged that the impairment of the homeostasis of mitochondria, the main energy generator in cells, is implicated in the pathogenesis/progression of IBD [16].

### 1.2. Mitochondrial Homeostasis

Mitochondria are crucial double-membrane organelles, involved in cellular energy production, Ca^2+^ signalling, transduction of stress and metabolic signals, induction of programmed cell death, and other cellular biosynthetic and oxidative functions [17]. Due to the endosymbiotic origin of mitochondria from ancient proteobacteria [18], mitochondria carry a small circular genome encoding 13 proteins (subunits of the respiratory complexes I, III-V of the OXPHOS (oxidative phosphorylation) system) and 22 tRNAs and 2 rRNAs (small 12S and Large 16S), required for proper translation of those proteins [19]. Despite many vital functions, mitochondria are also the main source of cellular ROS (reactive oxygen species) and RNS (reactive nitrogen species), which damage biomolecules (lipids, proteins, and DNA) and significantly increase the mutation rate of mtDNA (mitochondrial DNA). Thus, it is crucial to maintain mitochondrial homeostasis throughout the tight control of mitochondrial dynamics [20].

Mitochondria biogenesis is crucial for cellular homeostasis and includes the generation of new mitochondria, repair, and/or removal of damaged and malfunctional mitochondria or their parts. Mitochondria biogenesis is a complex process, requiring precise coordination between nuclear and mitochondrial genomes and could be influenced by many behavioural factors, cellular and environmental stresses (OS (oxidative stress), exercise, hypothermia, caloric restriction, and others) [21]. PGC1-α (peroxisome proliferator-activated receptor gamma coactivator 1-α) is a central regulator of mitochondrial biogenesis, which drives the cascade of other TFs (transcription factors) promoting transcription and replication of the mitochondrial genome (mainly via Tfam (mitochondrial transcription factor A)) [22].

Mitochondrial fusion and fission are key processes of mitochondrial dynamics, preserving central mitochondrial functions such as ATP generation, Ca^2+^ homeostasis, and involved in mitophagy, apoptosis, cell survival, and cell-cycle progression. Fusion is regulated by Mfm1 and Mfn2 (Mitofusin) proteins, located in the MOM (mitochondrial outer membrane), and Opa1 (optic atrophy 1), located in the MIM (mitochondrial inner membrane). Fusion allows mitochondria to buffer temporal stresses and compensate defects of each other, thereby generating functional mitochondria. Mitochondrial fission is aimed to isolate critically damaged components of mitochondria with the involvement of ER–mitochondria contact sites and cytosolic protein Drp1 (dynamin-related protein 1). Further damaged mitochondria are delivered to lysosomes for disposal, while healthy parts could reincorporate into the mitochondria network [20].

Individual severely damaged mitochondria are sent to degradation via the specialized form of autophagy—mitophagy, regulated by Pink1 (PTEN Induced Kinase 1) and Parkin (E3 Ubiquitin-Protein Ligase) proteins. In healthy mitochondria Pink1 is normally degraded; in damaged mitochondria, however, Pink1 is integrated into the MOM and recruits cytosolic Parkin. Further, Parkin leads to arrest of mitochondrial motility, thus putting it to quarantine and preventing accidental incorporation back into the mitochondrial network, and ubiquitinates proteins on the MOM, marking the mitochondria for lysosomal degradation [23].

Mitochondria have several quality control mechanisms to maintain cellular bioenergetic homeostasis. Mitochondrial matrix-localized chaperones promote protein folding and are required for protein import. Misfolded and improperly assembled protein complexes are recognized and degraded with matrix and MIM localized proteases [24]. While the majority of electron transport chain proteins are encoded by nuclear genes, they should be translated in the cytosol, transported into the mitochondria, and incorporated into targeted complexes with mitochondrial-encoded subunits. UPR^mt^ (mitochondrial unfolded protein response) is one of the protective mechanisms, monitoring mitochondrial homeostasis and preventing mitochondrial dysfunction by increasing the pool of mitochondrial quality control proteases and chaperones. Thus, UPR^mt^ is aimed to recover mitochondria that are possible to fix, whereas not salvageable organelles are targeted for degradation via mitophagy [25]. Mitochondrial function and UPR^mt^ are involved in the regulation of intestinal homeostasis, epithelial cell stemness, and differentiation, thus participating in the development of intestinal disease and contributing to the disease outcome [26].

Mitochondrial dysfunction (mainly the direct impact of mtDNA mutation/s, increased ROS production, and reduced ATP production) is closely associated with many neurogenerative and metabolic diseases [27].

### 1.3. IBD as a Risk Factor for Colorectal Cancer

CRC (colorectal cancer) is the third most frequently diagnosed malignancy, for both women and men, and the second most deadly type of cancer worldwide [28]. Its relationship with the chronic inflammatory process characteristic of IBD has been well defined [29]. Cytokines produced and secreted in the IBD-affected gut may both initiate the neoplastic process and promote cancer progression, especially by inhibiting apoptosis, promoting tumour growth, or facilitating metastasis [30]. Of these, TNF-α, which is responsible for maintaining chronic inflammation, also promotes tumour progression and angiogenesis. Additionally, activation of TNF-α induces the transcription factor NF-κB (nuclear factor-kappa B), which, in turn, is engaged in tumorigenesis of colorectal cancer and CAC (colitis-associated colorectal cancer). Of note, half of all CRCs and colitis-associated tumours show inappropriate activation of the NF-κB factor [31,32,33].

Another molecule involved in the development of colon cancer is IL-6. By activating JAK (Janus kinase), this pro-inflammatory cytokine contributes to the recruitment and activation of the oncogenic transcription factor—STAT3, which is involved, inter alia, in the process of cell division, cell survival, or apoptosis [34].

Although the formation of IBD-associated CRC appears to be similar to the development of sporadic CRC, there are undoubtedly differences in this process on the molecular level. In IBD patients, loss of function of *APC* (adenomatous polyposis coli), and key tumour suppressor genes, occurs less frequently and takes place later in the process of cancer development when compared to individuals with IBD-independent CRC. On the contrary, loss of function of p53, another tumour suppressor, which is essential for initiating cancer in IBD patients, occurs much earlier in these patients than in persons with sporadic CRC [35,36,37,38].

Macroscopically, specific risk factors for colon cancer development in IBD patients are the long duration and severity of IBD, the extensive colon coverage by the disease, as well as the presence of primary sclerosing cholangitis [39]. The maximum risk of developing colon cancer in UC patients is observed in those with pancolitis, while those with left-sided colitis have a moderate risk of developing colorectal carcinoma. In patients with CD, the increased risk of developing CRC is found in the case of lesions that affect 30–50% of the colon and starts increasing linearly after 6–8 years of the disease [39,40,41]. Despite the introduction of more effective immunosuppressive drugs, better diagnostic methods, and the more widespread use of colectomy as a method of eliminating high-grade dysplasia, it can still be assumed that the risk of CRC is doubled in IBD patients with a family history of colorectal cancer when compared to those without a family history of CRC [42,43].

## 2. Associated Mitochondrial Mutations and Dysfunctions

### 2.1. Mitochondrial Mutations Associated with IBD and Colorectal Cancer 

Polymorphisms in mtDNA are associated with different types of cancer by their effects on mtDNA copy number, mitochondrial ROS production, redox state, and release of mitochondrial intermediates [44]. MtDNA is highly variable, and different populations or ethnic groups may have a specific set of polymorphism sites and mutations, associated with a particular type of cancer. As so, such association was shown for colorectal cancer in Indians, Iranians, Polish, European Americans, and multi-ethnic cohorts [45,46,47,48,49,50,51].

A recent study has proved that mtDNA mutations accumulate and clonally expand in early tumorigenesis but afterwards are subject to purifying negative selection in cancer [52]. Accumulation and clonal expansion of mtDNA mutations in the healthy colon is known during ageing; however, a pathogenic condition such as IBD would accelerate this process due to the higher rate of cellular proliferation required for the epithelium regeneration [53]. This mechanism, when an increased rate of cellular proliferation would overload the replication system and lead to further mutations, is engaged in most cancers [54]. However, identified progression to malignancy was characterized by a decrease in the number and pathogenicity of mtDNA mutations, possible because of the outgrowth of one of the very low-frequency clones carrying non-pathogenic mtDNA mutations that shifted to homoplasmy [52]. Interestingly, a very close pattern of negative selection of mtDNA mutations was confirmed in other research, where similar sets of low-frequency mtDNA mutations were identified in normal and colorectal cancer samples [55]. The authors did not observe any association with age, gender, colorectal cancer stage, or tumour site with identified mtDNA mutations. It was proposed that the shift to glycolysis from oxidative phosphorylation in cancer cells may allow tolerance for accumulated mtDNA mutations because cancer cells are known to produce ATP mostly via glycolysis. However, this observation allows excluding the idea of the causative role of mtDNA mutations in colorectal cancer progression [55]. Thus, decreased mtDNA mutagenesis was identified in sporadic colorectal cancer and ulcerative colitis-associated cancer [56]. On the opposite, other research suggested the accumulation of mtDNA mutations in adenomatous polyps and CRCs, but with no influence of these mutations on their metabolic profiles [57]. Further functional studies are required to resolve the significance of the identified somatic mtDNA mutations on cancer phenotype and possible treatments. 

#### 2.1.1. Mitochondrial Energy Production in CRC Cells

The metabolic shift from OXPHOS to glycolysis, also called metabolic reprogramming, leads to amplification of glycolysis and mitochondrial retrograde signalling, allowing cancer cells to effectively adapt to the tissue microenvironment and speed up migration and invasion of cancer cells. However, due to the different energy output, a certain number of normal mitochondria are also important for prompt growth [58]. Many researchers have used COX (cytochrome c oxidase) complex to evaluate mitochondria status and define cancer progression biomarkers. mtDNA encodes 3 subunits of COX, and nuclear DNA encodes 10, forming complex IV of the ETC (electron transport chain) [59]. Further, it was suggested that the ratio of nucleus-encoded COX subunits to mtDNA-encoded subunits would increase during cancer progression [60]. Among other subunits, subunit IV plays a crucial role in the assembly of COX. However, recent studies have found no association between the levels of COX IV and colorectal cancer progression or prognosis of patients, while the COX IV was higher in female patients [61]. On the contrary, a comparison of primary colon cells with metastatic colorectal cancer cell lines has found higher mtDNA copy number and mitochondrial function in CRCs. In particular, higher levels of TFAM, COX-II, ND6 (NADH dehydrogenase subunit 6), and COX IV were detected in cancer in comparison to primary colon cells [62]. Similarly, a comparison of the mRNA levels of COX IV-1 and ATPase6 from patients with different stages of CRC have found that the decreased expression of COX IV-1 and ATPase6 correlates with increased ROS production during colorectal adenomatous polyp progression, thus pointing to the central role of COX IV-1 in the colorectal cell’s mitochondria energy production as they progress from polyps to carcinoma [63]. Other research has also confirmed higher and gender-specific expression of COX-II [64] and tumour grade-dependent of COX I [65].

However, we have to keep in mind that these studies have many differences in study design, ethnic background, population sampling, and experimental protocols that may affect observed results and different conclusions between different papers. Nevertheless, the role of COX in general and individual subunits in the development/progression of CRC and as a promising target for therapeutic interventions require further investigation.

#### 2.1.2. TRAP1 Functions in CRCs Mitochondrial Homeostasis and Metabolism

TRAP1 (Tumour Necrosis Factor Type 1 Receptor-Associated Protein) is an isoform of the HSP90 (heat shock protein) with mostly mitochondrial localization, known to be involved in colorectal carcinogenesis and with maximal expression at the transition point between low- and high-grade adenomas and in about 60–70% of human CRCs [66]. TRAP1 is co-upregulated in the majority of human CRCs altogether with its network of client/related proteins and, through them, involved in several central functions of cancer cells (bioenergetics, stemness, cell cycle progression, protection against cytotoxic agents and apoptosis, adaptation to stresses). Promising diagnostic and prognostic tools in human CRCs are reviewed in [67,68,69]. TRAP1 expression is higher in tumour tissues compared to surrounding non-malignant tissues, and elevation of TRAP1 protein levels and gene copy number correlate with malignant progression and metastasis of colorectal carcinoma [70,71,72]. TRAP1 is also known to affect mitochondrial architecture and dynamics, where TRAP1 knockdown favours mitochondrial fusion, while TRAP1 overexpression induces mitochondrial fission and subsequently enhanced migration in vitro and in tumour metastasis in vivo [73].

**Figure 1:**
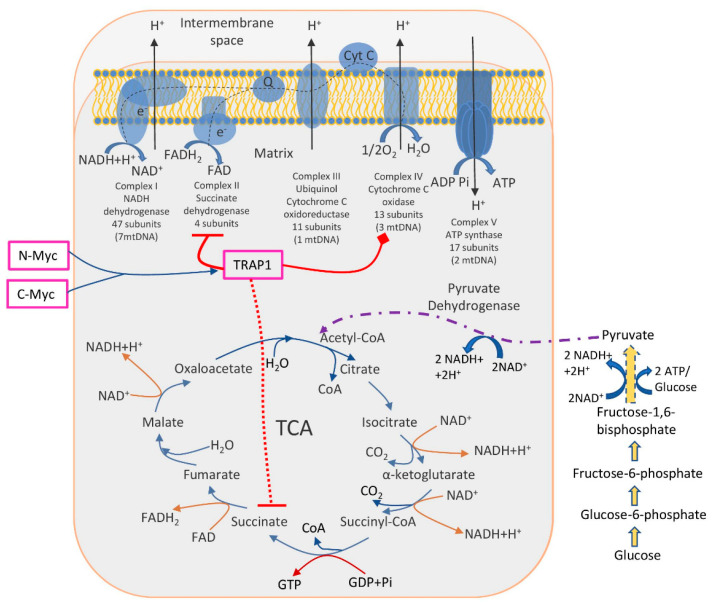
The role of TRAP1 in the regulation of metabolic switch between glycolysis and OXPHOS. TRAP1 was shown to inhibit OXPHOS (complexes II and IV, enzymes succinate dehydrogenase and cytochrome oxidase, respectively). Simultaneously, succinate dehydrogenase is a part of the TCA cycle; thus, its inhibition interrupts normal TCA functioning, further enhancing glucose utilization. Two oncogenic proteins N-Myc and C-Myc control TRAP1 expression and, subsequently, energy production in cancer cells.

Identified molecular pathways suggested that TRAP1 is involved in the switch toward aerobic glycolysis of tumours, i.e., decreased OXPHOS activity together with enhanced glucose utilization. The TRAP1 acts in two main directions: inhibits OXPHOS via downregulation of cytochrome oxidase (the complex IV of the respiratory chain) and inhibits SDH (succinate dehydrogenase), a metabolic enzyme that is a part of both the complex II of the respiratory chain and a component of the TCA cycle [74,75]. Another study has found that two oncogenic Myc proteins (c-Myc and N-Myc) transcriptionally control the expression of the TRAP1, thus regulating the proper folding and function of OXPHOS complex II and IV subunits, reducing ROS production, and enabling oxidative bioenergetics in tumour cells [76] (Figure 1).

Further, the crucial role of TRAP1 in balancing OXPHOS/glycolysis was shown in CRC patient-derived spheroids and cell lines, where TRAP1 enhances GLUT1 expression, glucose uptake, and lactate production and downregulates OXPHOS via interaction with glycolytic enzyme PFK1 (phosphofructokinase-1) [77]. Additionally, the involvement of ALDOB (B (Aldolase B, Fructose-Bisphosphate) and SLC16A4 (solute carrier family 16 members 4) in the regulation of the shift from OXPHOS to glycolysis was shown on CRC cell lines [57].

TRAP1 has a complex effect on both types of cells (healthy and tumour). Thus, further understanding the role of TRAP1 chaperone activity in oncogenic transition from healthy to tumour state under influence of different internal (genetic and metabolic) and external (toxins and environmental) factors would open a new line of possibilities to invent effective anti-CRC therapies and define innovative anti-neoplastic strategies.

### 2.2. Mitochondria Dysfunction

According to reports from the last several years, the disturbance of mitochondrial function may underlie the malfunctioning of the gastrointestinal tract and may even contribute to enteric inflammation [78]. Abnormalities in the structure and function of these energy-producing organelles have been observed in patients with inflammatory bowel diseases. Some of these abnormalities have been noticed selectively in individuals with a certain type of IBD or corresponding experimental models [13,79,80,81,82,83]. Others have been more generally described as IBD-related [84].

In the case of ulcerative colitis, a reduced function of mitochondrial respiratory chain complexes II, III, and IV (by 50–60%) has been reported in colonic biopsies from patients suffering from this disease [79]. However, the loss of MCJ (methylation-controlled J protein), an endogenous negative regulator of electron transport chain, proved to result in exacerbation of colitis in a murine experimental model of UC, suggesting that tight control of ETC is needed once the inflammatory process has started. The absence of MCJ additionally increased production of the intestinal permeability-related cytokines, such as IL-1β, and induced changes in gut microbiota composition, by affecting the levels of bile acids [80].

Perturbed mitochondrial dynamics have been observed in murine models of DSS (dextran sodium sulfate)-induced colitis. Increased levels of mRNA for the proteins involved in dynamic mitochondrial changes, such as Drp1, Fis1 (mitochondrial fission 1 protein), OPA1, MFN1, and MFN2, have been detected in colonic tissues of mice, suggesting a disturbance of mitochondrial fission and fusion processes in the course of colitis [81]. Colonic mitochondriopathy has also been observed in UC-suffering patients as a reduction in the expression of all 13 mtDNA-encoded genes that regulate ATP production [85].

Disturbances in β-oxidation of butyrate, the preferred source of energy of colonic epithelial cells, has also been linked to ulcerative colitis. As shown by Santhanam et al., the mitochondrial acetoacetyl CoA thiolase, an enzyme catalysing a critical step in butyrate oxidation, was impaired in the colonic mucosa of patients suffering from UC. Moreover, the authors concluded that this enzymatic defect might be triggered by increased mitochondrial ROS production [83]. Mitochondria-derived superoxide, in turn, has been reported to be the main initiator of the internalization and transcytosis of E. coli across epithelia in colonic biopsy specimens, as well as in cell lines. The epithelial barrier defect has been reduced by the administration of mitochondria-targeted antioxidants [86].

Jackson et al. reported the development of spontaneous ileal inflammation that was preceded by mitochondrial dysfunction due to the deletion of *Phb1* (prohibitin 1) in mice [13]. PHB1, being the major protein component of the inner mitochondrial membrane, participates in stabilizing proteins encoded by mitochondrial DNA or regulating the mitochondrial fusion process. It is also required to maintain the optimal activity of complexes I and IV of ETC [87]. Deletion of *Phb1* specifically in IEC (intestinal epithelial cells) resulted in the activation of UPR^mt^ and upregulation of Opa1, the major player in the process of mitochondrial fusion, in IEC. The results obtained by the authors may suggest that mitochondrial dysfunction preceded ileitis in the experimental model used. Likewise, deletion of *Phb1* in PC resulted in ileitis [13]. 

As it has been shown by Rath et al. [88], UPR^mt^ is activated in IEC from IBD-suffering patients, as well as in murine models of intestinal inflammation. Moreover, activation of UPR^mt^ due to IEC-specific loss of mitochondrial chaperon Hsp60 caused impaired mitochondrial respiration and the loss of stemness and cell proliferation in the intestinal crypts [89]. Similarly, ISC (intestinal stem cells)-specific deletion of mitochondrial *Hsp60*, and a consequent mitochondrial dysfunction, including diminished mitochondrial respiration, proved to be a trigger of a transition of ISC towards a PC-like phenotype. This confirmed the link between dysfunctional mitochondria and the loss of stemness observed in ileal CD [82]. Further details regarding the role of Hsp60 in the IBD development and progression, its use as a biomarker in the disease diagnosis, and a potential therapeutic target could be found in the recent review [90]. 

The participation of dysfunctional mitochondria in the pathophysiology of IBD has been also demonstrated by Heller et al. The authors proved that the reduced mitochondrial activity in the intestine activates the inflammation-associated pathways through an AMPK (AMP-activated protein kinase)-mediated manner. In the mechanism described, decreased mitochondrial activity due to selective inhibition of mitochondrial DNA polymerase in colon cells (ρ^0^ cells) results in activation of AMPKα2 and consequently facilitates NF-κB-dependent *IL-8* expression [84]. 

**Figure 2:**
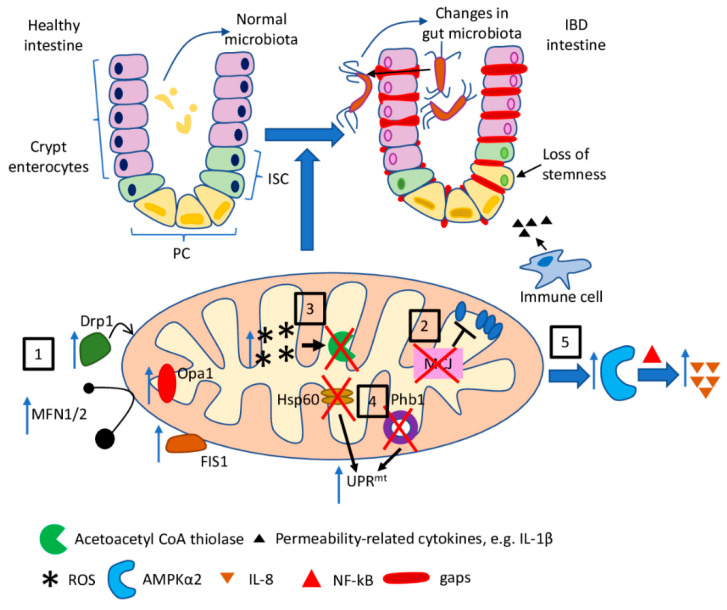
The role of mitochondria dysfunction in IBD. Mitochondria dysfunction observed in the course of inflammatory bowel diseases, as well as in experimental models of IBD, and the resultant features of the disease: (1) increased expression of fission- and fusion-related proteins (Fis1, Drp1, Opa1, MFN1/2) leads to disturbance in mitochondrial dynamics; (2) loss of MCJ results in the impaired regulation of ETC; (3) increased mitochondrial ROS production is a possible cause of the impairment of acetyl CoA thiolase leading to disturbances in mitochondrial β-oxidation of butyrate; (4) activation of UPR^mt^ (a) as a consequence of Phb1 deletion in IEC and PC and (b) due to IEC-specific loss of Hsp60 results in the loss of stemness in the intestinal crypts; (5) reduced mitochondrial activity leads to activation of AMPKα2 and subsequent NF-κB-dependent IL-8 expression.

Mitochondria-related molecular pathways discussed in this section are briefly summarised in Figure 2. 

### 2.3. Mitochondria-Related Pathways Identified in CRC

#### 2.3.1. NF-kB

Nuclear factor-kappa B is a crucial transcription regulator, which, upon activation, translocates into the nucleus where the expression of genes involved in a wide variety of biological functions are stimulated. NF-κB may be activated by different intra- and extra-cellular stimuli (bacterial and viral products, cytokines, ultraviolet irradiation, and radicals). NF-κB signalling is involved in immunity, cancer, inflammation, and nervous system function. Regarding mitochondria, NF-κB signalling was shown to participate in the regulation of mitochondria dynamics, respiration, gene expression, and metabolism (reviewed in [91]).

Recently, several researchers have described the involvement of NF-κB signalling in mitochondria dysfunction in CRC. As it was shown, the silencing of COX-1 leads to the depolarization of the mitochondrial membrane potential, inhibition of adenosine triphosphate production, increased generation of intracellular ROS, and triggered caspase-dependent mitochondrial apoptosis. Furthermore, COX-1 depletion inhibits NF-κB phosphorylation, which leads to the suppression of anti-apoptotic Bcl-2 and enhanced pro-apoptotic Bax protein expression. Thus, the role of COX-1 in NF-κB-mediated mitochondrial dysfunction and CRC progression is suggested [92]. Similarly, a novel mechanism connecting the role of mitochondrial dysfunction in tumour development and drug resistance was recently described. As it was shown on CRC-delivered mtDNA-depleted cell line, free calcium-dependent activation of NF-κB reduces the expression of tumour suppressor p53 [93]. ABCB7 (ABC transporter subfamily B member 7), one of the mitochondrial iron transporters regulating intracellular iron homeostasis, was shown to suppress apoptosis by inhibiting the expression of LDOC1 (an inhibitor of NF-κB) and to induce the hypoxia-independent accumulation of HIF1 (hypoxia-inducible factor 1). These results suggest that ABCB7 controls both apoptotic and non-apoptotic cell death and could be a novel target for CRC anticancer therapy [94].

#### 2.3.2. Reprogramming

OMA1 (OMA1 Zinc Metallopeptidase) is a well-known stress-activated mitochondrial protease, which promotes metabolic reprogramming and further CRC development. On the contrary, OMA1 knockout is known to suppress CRC development. Upon activation by hypoxia, OMA1 increases mitochondrial ROS to stabilize HIF-1α, thus promoting glycolysis and suppressing OXPHOS in CRC cells [95]. These results suggest the crucial role of OMA1 in HIF-1α-mediated CRC development and a high potential as a target for CRC therapy. Another nucleus-encoded mitochondrial membrane protein ANKRD22 (Ankyrin Repeat Domain 22) was shown to be activated by the tumour microenvironment and upregulated in colorectal cancer-initiating cells. ANKRD22 promotes glycolysis associated with a decrease in ATP/ADP and an increase in AMP/ATP levels. Acting via E-Syt1 (Extended Synaptotagmin-1), the lipid transport protein, ANKRD22 stimulates lipid transport into mitochondria and reduces the number of mitochondria, thus further promoting the reprogramming of cancer cells to meet their metabolic requirements [96].

#### 2.3.3. Protein Quality Control

HSP60 is a mitochondrial chaperone responsible for maintaining mitochondria proteostasis and is highly expressed in tumours compared to healthy cells, thus suggesting that HSP60 expression could be beneficial for tumour growth. Indeed, HSP60 knock-down resulted in inhibited cell proliferation via disrupted mitochondrial homeostasis. On the molecular level, HSP60 knockdown causes an increase in the cellular adenine levels with subsequent activation of the AMPK pathway. Further, AMPK is an inhibitor for mTOR-mediated protein synthesis, resulting in a decreased speed of cell proliferation [97].

#### 2.3.4. PGC-1α

PGC-1α (peroxisome proliferator-activated receptor gamma coactivator 1-alpha) is a TF highly expressed in the mitochondria and tissues and regulates energy metabolism, mitochondrial biogenesis, homeostasis, and other biological functions [98]. PGC-1α is also involved in cancer progression, proliferation, invasion, and several metabolic pathways, responsible for drug resistance in diverse cancers [99]. As it was recently shown, 5FU (5-fluorouracil)-resistant CRC cells have increased PGC-1α expression, resulting in the absence of a significant decrease in the mitochondrial biogenesis or activities of mitochondrial complex I and IV, as well as a weak decrease in the antioxidant enzymatic activity, cell survival, and oxygen consumption ratio. PGC-1α in the 5FU-resistant CRC cells was shown to inhibit ER-stress and suppress apoptosis [100]. Hypoxia also increases the expression of PGC-1α and decreases ROS production. Similarly, up-regulation of PGC-1α was associated with increased resistance to the anti-cancer drug 5FU and enhanced proliferation, sphere formation, and motility of CRC [101]. SIRT3 (Sirtuin 3, NAD-Dependent Protein Deacetylase Sirtuin-3, Mitochondrial) is a crucial mitochondrial protein, known to eliminate ROS, inhibit apoptosis, and prevent the formation of cancer cells [102,103]. It was found that SIRT3 expression affects CRC cell sensitivity to chemotherapy, acting via SOD2 (superoxide dismutase 2) and PGC-1α-mediated pathways [104]. SIRT3 expression is associated with mitochondrial ROS levels and apoptosis induction in CRC cells treated with anti-cancer drugs. SIRT3 suppression leads to increased mitochondrial ROS production, decreased PGC-1α expression and mitochondrial function, and, subsequently, to higher sensitivity to anti-cancer drugs. On the other side, SIRT3 knock-down leads to decreased SOD2 expression and activity, decreased mitochondrial activity, and increased apoptosis, with further improved sensitivity to anti-cancer drugs [104].

#### 2.3.5. IGF-1R

IGF-1R (insulin-like growth factor receptor) is one of the key molecular hubs where several major signalling pathways involved in human physiology and pathophysiology are converged. Many pieces of evidence have indicated that an increased level of IGF-1R is associated with cell survival and proliferation, metastasis and cancer progression, anti-cancer drug resistance, and poor prognosis for patients [105,106]. As it was recently shown, IGF-1R acts via LKB1/AMPK pathways at the nexus between oxidative damage, mitochondrial function, and a connection between colitis and colorectal cancer. Mechanically, heterozygous IGF-1R knock-out attenuated colitis and CAC, induced in Igf1r^+/−^ mice. Igf1r^+/−^ cells were protected from oxidative stress via an improved biological function of mitochondrial fusion, increased respiratory coupling index, oxidative phosphorylation index, oxygen consumption rate, and decreased extracellular acidification rate [107]. An additional molecular mechanism of action, identified in heterozygous Igf1r^+/−^ mice, was mediated via IGF-1R knockdown-triggered increase in MDA5 and RIG-I expression. These results were confirmed with silenced IGF-1R in normal and colonic cancer human cells. *MDA5* (melanoma differentiation-associated gene 5), an intracellular sensor of viral RNA that triggers the innate immune response and *RIG-I* (Retinoic Acid-Inducible Gene I Protein), is involved in viral double-stranded RNA recognition, regulation of the antiviral innate immune response, and acted in PI3K-Akt-independent pathways, thus suggesting a new signal transduction pathway, leading to MDA5- and RIG-I-mediated mitochondrial apoptosis in cancer cells [108].

#### 2.3.6. Mitochondrial Dynamics

Further, the vital role of mitochondrial dynamics was proven in several studies, and treatments with mitophagy stimulation drugs were proven effective on different types of cancer [23]. Application of drugs selectively targeting mitochondria and inducing mitophagy in cancer cells, including Mito-CP (3-carboxyl proxyl nitroxide) and Mito-Metformin, resulted in depleted levels of intracellular ATP and persistently inhibited ATP-linked oxygen consumption in colon cancer cells. The molecular signalling pathway of these drugs relies on the activation of the AMPK pathway, suppression of the mTOR target RPS6KB1 (ribosomal protein S6 kinase B1), and release of ULK1 (Unc-51-like autophagy-activating kinase 1) from mTOR-mediated inhibition. In particular, Mito-CP and Mito-Metformin were effective in colon cancer cells carrying the KRAS proto-oncogene mutation and had limited effect on non-transformed intestinal epithelial cells [109]. 

The Hippo-Yap pathway is involved in development, growth, repair, and homeostasis, but it is also involved in the development and progression of multiple cancers [110]. As it was recently shown in human rectal cancer cells, Hippo-Yap could act as a tumour promoter via restricting JNK-Drp1-mediated mitochondrial fission. Yap is upregulated in CRC cells and positively correlates with cell survival and migration. However, Yap silencing promotes JNK phosphorylation with further Drp1 activation and translocation to the mitochondria, thus initiating mitochondrial fission. Excessive mitochondrial fission triggers cellular apoptosis and leads to impaired cellular migration and invasion [111]. A similar role was shown also for the related Hippo-Mst1 (Macrophage Stimulating 1) pathway, where Mst1 plays a crucial role in colorectal cancer stress response involving regulation of mitophagy via JNK/p53/Bnip3 pathways. CRC cells have down-regulated Mst1, while Mst1 overexpression induces CRC cells apoptosis and impairs proliferation and migration [112]. Thus, mitophagy-targeted therapy may be a new approach in CRC treatment.

The discussed connection between mitochondrial dysfunctions and IBD/CRC are briefly summarized with major involved pathways in Table 1.

## 3. Mitochondria-Targeted Therapy as an IBD Treatment and CRC Prevention—Points of Possible Intervention

IBD is a chronic and incurable disease, and the nature of its pathomechanism may favour its progression to colorectal cancer [30]. Although current therapies to eliminate/minimize bothersome and even life-threatening symptoms have improved significantly over the past few years, they remain expensive and not always effective [113,114]. Additionally, most of them target immune mechanisms, resulting in an increased incidence of opportunistic infections in patients [115]. Recent discoveries linking mitochondrial dysfunction/mtDNA mutations with the pathophysiology of inflammatory bowel diseases have opened the door to a search for new, promising treatments for IBD, targeting an early inducer of inflammation [2].

Most of the recently proposed, or tested, mitochondria-targeted IBD therapies have focused on eliminating mitochondria-derived ROS [116]. mtROS are produced in an increased amount by dysfunctional mitochondria, causing tissue damage and mediating stress signalling [117,118]. It has been proven that the use of mitoTEMPO, a specific scavenger of mitochondrial superoxide, not only sealed the epithelial barrier and reduced the severity of the disease in mice with DSS-induced colitis [86], but it also improved mitochondrial ultrastructure and ameliorated UPR^mt^ and UPR^ER^ responses as well as PC abnormalities in mice with tamoxifen-induced *Phb1* deletion. Additionally, mitoTEMPO improved the viability and minimized the defects of PC in intestinal enteroids derived from the crypts of *Phb1*^iΔIEC^ and *Phb1*^ΔPC^ mice [13]. The analysis of the mRNA transcriptome in terminal ileal mucosal biopsies from type I CD-suffering patients (with PC defects), as well as in non-IBD individuals, revealed that the use of mitoTEMPO normalized the expression of *IL-17*/*IL-23* and genes associated with apoptosis and lipid metabolism, compared to healthy individuals [119]. The use of an antioxidant MitoQ, a derivative of coenzyme Q, to block the damaging effects of mtROS in people with moderate UC, is currently a subject of a randomized phase 2b double-blind placebo-controlled trial. The idea of the MARVEL trial (Mitochondrial Anti-oxidant Therapy to Resolve Inflammation in Ulcerative Colitis) is to administer MitoQ (or placebo) tablets to patients with active UC flare-up, in addition to standard treatment, to resolve the inflammation process at the moment it begins [120].

Another proposition of mitochondria-targeted IBD therapy involves the enhancement of mitochondrial respiration. A study presented by Khaloian et al. tested the possibility of reversing the inflammation-associated growth defect of crypts derived from TNF^ΔARE^ mice—a model of chronic CD-like ileitis. The authors showed that the treatment of the crypts with dichloroacetae, a selective inhibitor of pyruvate dehydrogenase kinase, restored the stemness and allowed the organoids to grow in culture, by improving the mitochondrial respiration [82].

Finally, targeting excessive mitochondrial fission, which is one of the components of enteric inflammation, is a promising strategy for fighting IBD. Mancini et al. tested the efficacy of P110, a selective inhibitor of Drp1-Fis1-driven mitochondrial fission, in murine models of colitis. The researchers proved that the systemic administration of the inhibitor decreased the severity of chemically evoked colitis in mice. Additionally, DSS-induced disturbances in mitochondrial energetics and fragmentation in mouse epithelial cell lines and bone marrow-derived macrophages were mitigated by the application of P110 [81].

Further understanding of the pathomechanism of inflammatory bowel diseases, including the role of mitochondrial dysfunction/mtDNA mutations in the development and progression of IBD, will certainly allow for the invention of more target-oriented and effective therapies for this group of disorders. This, in turn, will prevent the malignant transformation of inflamed tissues.

## 4. Conclusions

The close association between IBD and CRC has been proved by many researchers. In addition to known genetic and environmental factors, mitochondrial homeostasis is also one of the key factors involved in IBD development and its progression to CRC. Certain mitochondrial mutations and dysfunctions were linked with IBD and could serve as biomarkers for CRC. In the current review, we have discussed main players in the mitochondria-related molecular pathways (such as *NF-kB*, *PGC-1α*, *IGF-1R*, *TRAP1*, *Phb1*, and others) and their potential as targets for mitochondria-based treatments. However, further research is required for a better understanding of the role of mitochondria and mitochondria-localized proteins in IBD and CRC development, as well as the identification of more effective targets for pharmacological intervention and therapies. 

## Figures and Tables

**Table 1:** The role of mitochondrial/mitochondria-localized proteins in IBD and CRC.

Cell Line/Gene	Effect	Reference
*COX-1*	Silencing of *COX-1* leads to mitochondrial depolarization, inhibition of ATP production, increased ROS, and triggers caspase-dependent mitochondrial apoptosis. COX-1 depletion inhibits NF-κB phosphorylation and further suppression of anti-apoptotic Bcl-2 and enhanced pro-apoptotic Bax protein expression.	[92]
MtDNA-depleted CRC-delivered cell line	Free calcium-dependent activation of NF-κB reduces the expression of tumour suppressor p53.	[93]
*ABCB7*	ABCB7 suppresses apoptosis by inhibiting the expression of LDOC1 (an inhibitor of NF-κB) and induces HIF-1α accumulation.	[94]
*OMA1*	OMA1 increases mitochondrial ROS to stabilize HIF-1α, thus promoting glycolysis and suppressing OXPHOS in CRC cells. OMA1 knockout is known to suppress CRC development.	[95]
*ANKRD22*	ANKRD22 promotes glycolysis associated with a decrease in ATP/ADP and an increase in AMP/ATP levels. Acting via E-Syt1, ANKRD22 stimulates lipid transport into mitochondria and reduces the number of mitochondria.	[96]
*HSP60*	HSP60 knock-down resulted in inhibited cell proliferation via disrupted mitochondrial homeostasis and increase in the cellular adenine levels with subsequent activation of the AMPK pathway. Further, AMPK is an inhibitor for mTOR-mediated protein synthesis, resulting in a decreased speed of cell proliferation.	[97]
*PGC-1α*	5FU-resistant CRC cells have increased PGC-1α expression, resulting in the absence of a significant decrease in the mitochondrial biogenesis or activities of mitochondrial complex I and IV as well as a weak decrease in the antioxidant enzymatic activity, cell survival, and oxygen consumption ratio. PGC-1α in the 5FU-resistant CRC cells was shown to inhibit ER stress and suppress apoptosis.	[100]
	Hypoxia increases the expression of PGC-1α and decreases ROS production. Up-regulation of PGC-1α was associated with increased resistance to 5FU and enhanced proliferation, sphere formation, and motility of CRC cells.	[101]
	SIRT3 expression affects CRC cell sensitivity to chemotherapy via SOD2 and PGC-1α-mediated pathways. SIRT3 expression is associated with mitochondrial ROS level and apoptosis induction in CRC cells treated with anti-cancer drugs. SIRT3 suppression leads to increased mitochondrial ROS production, decreased PGC-1α expression and mitochondrial function, and, subsequently, to higher sensitivity to anti-cancer drugs. SIRT3 knock-down leads to decreased SOD2 expression and activity, decreased mitochondrial activity, and increased apoptosis, with further improved sensitivity to anti-cancer drugs.	[104]
*IGF-1R*	Heterozygous IGF-1R knock-out attenuated colitis and CAC induced in Igf1r^+/−^ mice. Igf1r^+/−^ cells were protected from OS via an improved biological function of mitochondrial fusion, increased respiratory coupling index, oxidative phosphorylation index, oxygen consumption rate, and decreased extracellular acidification rate.	[107]
	IGF-1R knock-down triggered an increase in MDA5 and RIG-I expression. MDA5 and RIG-I act in PI3K-Akt-independent pathways, suggesting a new signal transduction pathway, leading to MDA5- and RIG-I-mediated mitochondrial apoptosis in cancer cells.	[108]
Mito-CP and Mito-Metformin	Mitophagy-inducing drugs Mito-CP and Mito-Metformin lead to depleted levels of intracellular ATP and persistently inhibits ATP-linked oxygen consumption in CRC cells. These drugs activate the AMPK pathway, suppress the mTOR target RPS6KB1, and release ULK1 from mTOR-mediated inhibition.	[109]
Hippo-Yap	Hippo-Yap acts as a tumour promoter via restricting JNK-Drp1-mediated mitochondrial fission. Yap is up-regulated in CRC cells and positively correlates with cell survival and migration. Yap silencing promotes JNK phosphorylation with further Drp1 activation and initiation of mitochondrial fission, which triggers cellular apoptosis and leads to impaired cellular migration and invasion.	[111]
Hippo-Mst1	Mst1, as a part of the Hippo-Mst1 pathway, regulates mitophagy via JNK/p53/Bnip3 pathways. CRC cells have down-regulated Mst1, while Mst1 overexpression induces CRC cell apoptosis and impairs proliferation and migration.	[112]

## Data Availability

Not applicable.

## Abbreviations

5FU	5-fluorouracil
ABCB7	ABC transporter subfamily B member 7
ALDOB	Aldolase B, Fructose-Bisphosphate
AMPK	AMP-activated protein kinase
ANKRD22	Ankyrin Repeat Domain 22
APC	adenomatous polyposis coli
CAC	colitis-associated colorectal cancer
CD	Crohn’s disease
COX	cytochrome c oxidase
CRC	colorectal cancer
Drp1	dynamin-related protein 1
DSS	dextran sodium sulfate
E-Syt1	Extended Synaptotagmin-1
ETC	electron transport chain
Fis1	mitochondrial fission 1 protein
GWAS	genome-wide association studies
HIF1	hypoxia-inducible factor 1
HSP	heat shock protein
IBD	inflammatory bowel diseases
IGF-1R	Insulin-like growth factor receptor
IL	interleukin
ISC	intestinal stem cells
JAK	Janus kinase
MARVEL	Mitochondrial Anti-oxidant Therapy to Resolve Inflammation in Ulcerative Colitis
MCJ	methylation-controlled J protein
MDA5	melanoma differentiation-associated gene 5
Mfn	mitofusin
MIM	mitochondrial inner membrane
Mito-CP	3-Carboxyl proxyl nitroxide
MOM	mitochondrial outer membrane
Mst1	Macrophage Stimulating 1
mtDNA	mitochondrial DNA
ND6	NADH dehydrogenase subunit 6
NOD2	nucleotide-binding oligomerization domain-containing protein 2
NF-κB	nuclear factor-kappa B
OMA1	OMA1 Zinc Metallopeptidase
Opa1	optic atrophy 1
OS	oxidative stress
OXPHOS	oxidative phosphorylation
Parkin	E3 Ubiquitin-Protein Ligase
PC	Paneth cells
PFK1	phosphofructokinase-1
PGC-1α	Peroxisome proliferator-activated receptor gamma coactivator 1-alpha
Phb1	prohibitin 1
Pink1	PTEN Induced Kinase 1
RIG-I	Retinoic Acid-Inducible Gene I Protein
RNS	reactive nitrogen species
ROS	reactive oxygen species
RPS6KB1	ribosomal protein S6 kinase B1
SDH	succinate dehydrogenase
SIRT3	Sirtuin 3, NAD-Dependent Protein Deacetylase Sirtuin-3, Mitochondrial
SLC16A4	solute carrier family 16 members 4
SOD2	superoxide dismutase 2
Tfam	mitochondrial transcription factor A
TFs	transcription factors
TNF-a	tumour necrosis factor alpha
TRAP1	Tumour Necrosis Factor Type 1 Receptor-Associated Protein
UC	ulcerative colitis
ULK1	Unc-51-like autophagy-activating kinase 1
UPR^mt^	mitochondrial unfolded protein response
